# Elucidating *Thermothielavioides terrestris* secretome changes for improved saccharification of mild steam-pretreated spruce

**DOI:** 10.1186/s13068-024-02569-3

**Published:** 2024-10-05

**Authors:** Fabio Caputo, Romanos Siaperas, Camila Dias, Efstratios Nikolaivits, Lisbeth Olsson

**Affiliations:** 1 Division of Industrial Biotechnology, Department of Life Sciences, Chalmers University of Technology, Kemivägen 10, 412 96 Gothenburg, Sweden; 2 Present Address: Industrial Biotechnology & Biocatalysis Group, Biotechnology Laboratory, School of Chemical Engineering, National Technical University of Athens, Heroon Polytechniou 9, 15772 Athens, Greece; 3 Wallenberg Wood Science Center, Chalmers University of Technology, Kemigården 4, 412 96 Gothenburg, Sweden

**Keywords:** Steam-explosion, Lignocellulose mild pretreatment, Spruce saccharification, Secretomics

## Abstract

**Background:**

The efficient use of softwood in biorefineries is hampered by its recalcitrance to enzymatic saccharification. In the present study, the fungus *Thermothielavioides terrestris* LPH172 was cultivated on three steam-pretreated spruce materials (STEX_180°C/auto_, STEX_210°C/auto_, and STEX_210°C/H2SO4_), characterized by different hemicellulose content and structure, as well as on untreated biomass. The aim of the study was to map substrate-induced changes in the secretome of *T. terrestris* grown on differently treated spruce materials and to evaluate the hydrolytic efficiency of the secretome as supplement for a commercial enzyme mixture.

**Results:**

The cultivation of *T. terrestris* was monitored by endo-cellulase, endo-xylanase, endo-mannanase, laccase, and peroxidase activity measurements. Proteomic analysis was performed on the secretomes induced by the spruce materials to map the differences in enzyme production. Growth of *T. terrestris* on STEX_180°C/auto_ and STEX_210°C/auto_ induced higher expression level of mannanases and mannosidases of the GH5_7 CAZy family compared to cultivation on the other materials. Cultivation on untreated biomass led to overexpression of GH47, GH76, and several hemicellulose debranching enzymes compared to the cultivation on the pretreated materials. *T. terrestris* grown on untreated, STEX_180°C/auto_ and STEX_210°C/auto_ induced three arabinofuranosidases of the GH43 and GH62 families; while growth on STEX_210°C/H2SO4_ induced a GH51 arabinofuranosidase and a GH115 glucuronidase. All secretomes contained five lytic polysaccharide monooxygenases of the AA9 family. Supplementation of Celluclast® + Novozym188 with the secretome obtained by growing the fungus grown on STEX_180°C/auto_ achieved a twofold higher release of mannose from spruce steam-pretreated with acetic acid as catalyst, compared to the commercial enzyme cocktail alone.

**Conclusions:**

Minor changes in the structure and composition of spruce affect the composition of fungal secretomes, with differences in some classes explaining an increased hydrolytic efficiency. As demonstrated here, saccharification of spruce biomass with commercial enzyme cocktails can be further enhanced by supplementation with tailor-made secretomes.

**Supplementary Information:**

The online version contains supplementary material available at 10.1186/s13068-024-02569-3.

## Background

The European Commission's biorefinery report indicates that the circular bioeconomy could help reduce greenhouse gas emissions by up to 55% within 2030 if biorefineries were implemented on a wider scale [1]. However, for that to happen, several technical challenges must be overcome, including the efficiencies of hydrolytic enzymes [1, 2]. The saccharification step represents the second main operational cost for a biorefinery, after biomass pretreatment (~ 25–30% of the costs) [3]. The high cost is partially due to the limited availability of efficient enzyme mixtures. Hence, screening for enzymes that can improve saccharification efficiency has become of paramount importance. Furthermore, given the ample range of raw materials suitable for biorefinery application, it is important that the enzymes mixtures are tailored to the raw material.

Filamentous fungi can grow on any organic matter. They rely on their extracellular protein machinery to acquire nutrients, construct and remodel the cell wall, and compete with other organisms [4]. Filamentous fungi adjust their secretome in response to the surroundings and adapt enzyme expression by means of a signaling and regulatory network. Consequently, they can adapt their metabolism in response to changes in the environment, such as the availability of carbon and nitrogen sources [4–7]. In general, the availability, quality, and complexity of the carbon source influences secretome composition [6]. Different studies have examined the influence of plant composition on expression and secretion of extracellular enzymes by wood-degrading fungi [8–10]. Therefore, filamentous fungi can be exploited to produce substrate-specific and more efficient enzyme mixtures for lignocellulosic biomass saccharification [3, 11, 12].

In the present work, *Thermothielavioides terrestris* LPH172 was grown on differently steam-pretreated spruce to determine the influence of substrate pretreatment on the fungal secretome. *T. terrestris* was selected due to its thermophilic nature and availability of a sequenced and annotated genome, indicating a large potential for lignocellulosic biomass deconstruction. The genome of *T. terrestris* LPH172 harbors 411 individual Carbohydrate Active enZyme (CAZy) genes, categorized in 201 glycosyl hydrolases (GHs), 86 glycosyl transferases, 4 polysaccharide lyases, 26 carbohydrate esterases (CEs), 83 auxiliary activity (AA) enzymes, and 11 carbohydrate-binding modules (CBMs) [13].

Steam-pretreated spruce was selected as a substrate due to its industrial relevance as a raw material for biorefineries, its abundance, easy access in boreal forests, and sustainable exploitation in Sweden, being a fundament in their bioeconomy [14]. Steam pretreatment (STEX) offers a mature and flexible technology for the pretreatment of lignocellulosic biomass. However, in spruce, it requires the addition of acids to compensate for the few acidic groups present only on galactoglucomannan (1 acetyl group every 3–4 hexose units) [15–17]. Addition of a significant amount of acid during pretreatment contributes to hemicellulose solubilization and the formation of secondary degradation compounds, but these may inhibit microbial fermentation [18, 19].

The aim of the present work was to map substrate-induced changes in the secretome of *T. terrestris* grown on differently steam-pretreated spruce, and to evaluate the hydrolytic efficiency of the secretome as supplement for a commercial enzyme mixture. Based on a prior study [20], pretreatment conditions included two different temperatures (180 °C and 210 °C), with and without the addition of 0.1%(w/w) sulphuric acid (H_2_SO_4_), denoted as STEX_180°C/auto_, STEX_210°C/auto_, STEX_210°C/H2SO4,_ respectively. Thus, using different pretreatment conditions, the fungus was introduced to spruce with diverse hemicellulose content and structural characteristics. Particularly, cellulose microfibrils reorganized during pretreatment, leading to the formation of larger microfibril aggregates. This microfibril rearrangement likely contributed to the observed increase in enzymatic hydrolysis yields as it improved enzyme accessibility. Lignin was present in all materials. *T. terrestris* was grown for 14 days, during which its growth was monitored by enzyme activity measurements. Differences in enzyme secretion obtained from the fungal growth on untreated and steam-pretreated spruce were assessed by proteomic analysis. The hydrolytic efficiency of the enzymes secreted by *T. terrestris* was further evaluated upon supplementation of the cellulolytic cocktail Celluclast® + Novozym188.

## Materials and methods

### Pretreatment and fungal carbon source selection

Spruce used in this work was steam-pretreated as described in our previous work [20]. The selected conditions included two different temperatures (180 °C and 210 °C), with and without the addition of 0.1% (w/w) H_2_SO_4_, denoted as STEX_180°C/auto_, STEX_210°C/auto_, and STEX_210°C/H2SO4_. The material used for enzymatic hydrolysis, denoted as STEX_210°C/HAc_, was steam-pretreated at 210 °C with 1% (w/w) acetic acid [20]. Composition of the raw and steam-pretreated materials was determined in triplicates according to National Renewable Energy Laboratory standard methods [21], and is presented in the supplementary material (Table S1). Except for STEX_210°C/Hac_, the materials used in this work were milled for 30 s in an IKAA10S knife mill (IKA®-Werke GmbH & Co. KG, Staufen, Germany), after which the dry mass (DM) was calculated based on the average of four different moisture measurements (VWR, Radnor, PA, USA).

The fungal strain *T. terrestris* LPH172 (previously *Thielavia terrestris*) was kept at 4 °C on potato dextrose agar (PDA) plates. Spores were collected in phosphate-buffered solution (PBS) + 1% (v/v) Tween20, from PDA plates grown at 45 °C for 5 days. After collection, spores were counted under the light microscope using a hemocytometer. The base medium used for liquid cultures contained 4.0 g L^−1^ KH_2_PO_4_, 13.6 g L^−1^ (NH_4_)_2_SO_4_, 0.8 g L^−1^ CaCl_2_·2H_2_O, 0.6 g L^−1^ MgSO_4_·7H_2_O, 0.1 g L^−1^ peptone (Gibco BRL, Paisley, Scotland), and 0.1 g L^−1^ yeast extract (VWR Chemicals BDH, Prolabo, Australia) supplemented with 1000 × trace element solution (3.0 g L^−1^ FeSO_4_·7H_2_O, 4.5 g L^−1^ ZnSO_4_·7H_2_O, 4.5 g L^−1^ CaCl_2_·2H_2_O, 1.0 g L^−1^ MnCl_2_·4H_2_O, 0.3 g L^−1^ CoCl_2_·6H_2_O, 0.3 g L^−1^ CuSO_4_·5H_2_O, 0.4 g L^−1^ Na_2_MoO_4_·2H_2_O, 1.0 g L^−1^ H_3_BO_3_, 0.1 g L^−1^ KI, and 19.0 g L^−1^ C_10_H_14_N_2_Na_2_O_8_ 2H_2_O) and set at pH 5.6.

### Enzyme assays, protein quantification, and total fungal biomass

Endo-cellulase, endo-xylanase, endo-mannanase, laccase and peroxidase were measured on the secreted enzymes from *T. terrestris*. Filter paper unit, beta-glucosidase, xylanase, and mannanase were measured in the commercial mixtures. The reason for choosing two different sets of enzymatic assays was due to that methods relying on DNS detection (filter paper unit, xylanase, and mannanase activity assays) were not suitable for the complex sample matrix in the secretome resulting in low sensitivity. Protein quantification was performed with the Bradford assay.

#### Endo-cellulase, endo-xylanase, and endo-mannanase activity

Endo-cellulase, endo-xylanase, and endo-mannanase activity was assayed in the same way, but with different substrates: Azo-CM-Cellulose, Azo-Xylan, and Azo-Carob Galactomannan, respectively (Megazyme, Bray, Ireland). Briefly, 2 g of each substrate was dissolved in 80 mL milliQ water and stirred for 20 min on a hot plate. Then, 5 mL of sodium acetate buffer (2 M, pH 4.5) were added, the pH was adjusted to 4.5, and the volume was adjusted to 100 mL with milliQ water. For the enzymatic assay, 125 µL sample were added to 125 µL substrate and incubated for 10 min at 40 °C using a thermomixer set at 600 rpm (Eppendorf, Hamburg, Germany). The reactions were stopped by adding 625 µL precipitant solution (200 mL of 200 g L^−1^ CH_3_COONa·3H_2_O and 20 g L^−1^ (CH_3_CO_2_)_2_Zn mixed with 800 mL of 95% ethanol). After 10 min of equilibration at room temperature, the reactions were centrifuged for 3 min at 4000*g* and 200 µL of the supernatant were transferred to a 96-well plate. Absorbance was measured at 590 nm against a blank (buffer + substrate) with a plate reader (BMG LABTECH GmbH, Ortenberg, Germany).

#### Laccase and peroxidase activity

Laccase and peroxidase activity was measured in 96-well plates in a 200-µL reaction volume. For laccase, the reaction mixture contained 1.5 mM 2,2'-azino-bis(3-ethylbenzothiazoline-6-sulfonic acid) in 50 mM citrate buffer pH 4.5 [22]; for peroxidase, it contained also 10 mM H_2_O_2_ [23]. The activity measurements in the presence of H_2_O_2_ would be considered as the sum of laccase and peroxidase activities. Plates were incubated at 40 °C for 15 min and absorbance was measured at 420 nm.

#### Bradford assay

Protein content was measured with the Bradford method [24] following precipitation. Briefly, 200 µL of sample, 20 µL of 500 mM K_3_PO_4_, 20 µL of 250 mM CaCl_2_, and 500 µL of pure ethanol were added in a 1.5-mL Eppendorf tube. After mixing, the samples were centrifuged at 14,000 rpm for 1 min and the supernatant was removed. The pellet was incubated with 200 µL undiluted 5 × Bradford reagent for 10 min, 800 µL of water were added, and absorbance was read at 595 nm.

#### Enzymatic activity measurements of Celluclast.® and Novozym188

Different assays were used to measure the enzymatic activity in Celluclast® and Novozym188. Particularly, Celluclast® was analyzed for cellulolytic and beta-glucosidase activity; Novozym188 for beta-glucosidase activity.

Cellulolytic activity was measured using the filter paper unit assay [25] with some adjustments [5]. Beta glucosidase activity was measured using the substrate p-nitrophenyl-β-D-glucopyranoside as reported elsewhere [26] with some adjustments [20]. Xylanase and mannanase activity were measured as described elsewhere [27], with the release of reducing sugars from xylan and glucomannan quantified by the 3,5-dinitrosalicylic acid method [25].

### Screening for sugars as CAZyme inducers

Different sugars (glucose, xylose, arabinose, cellobiose, and maltose) were evaluated as inducers of CAZyme activities in *T. terrestris* grown in basal medium*.* Monosaccharides and disaccharides were supplemented to FBM + trace metals solution (details reported in "Pretreatment and fungal carbon source selection" section) at a concentration of 0.056 M and 0.028 M, respectively. Briefly, 20 mL of prepared media were inoculated with 10^6^ spores. The experiment was performed in biological triplicates incubated at 45 °C under shaking (130 rpm) for 6 days. Endo-cellulase, endo-xylanase, and endo-mannanase activities were measured on days 3, 4, and 6, and final biomass was measured on day 6 (see section S1).

### Time-course of *T. terrestris* growth on spruce via enzymatic assays

Autoclaved basal medium (150 mL) supplemented with 2% (DM) w/v of each spruce material (autoclaved beforehand) was inoculated with 15 mL T*. terrestris* biomass pregrown on arabinose. The suspension was incubated at 45 °C and 130 rpm for 14 days. Every other day, 1.4 mL from each flask were sampled using wide-orifice tips. The experiment was performed in biological triplicates and activity measurements were performed in technical triplicates. Enzymatic activity (endo-cellulase, endo-xylanase, endo-mannanase, laccase, and peroxidase) was measured right after sampling; whereas protein quantification was carried out with samples stored at – 20 ºC.

### Preparation of samples for proteomic analysis

For the proteomic analysis of secretomes obtained upon growth on each material, fermentation was stopped 4 days after inoculation by centrifuging the culture medium at 10,000*g* for 10 min. Culture supernatants free of spruce material and fungal biomass, were filtered through a 0.2-μm pore size filter and then concentrated 5- to 10-fold in Jumbosep™ centrifugal inserts (Pall, Port Washington, NY, USA). The concentrated supernatants were dialyzed against PBS overnight at 4 °C and then freeze-dried.

### Functional annotation of proteins

Proteins were annotated with UniFire (release 2023.1). CAZymes were identified with BlastP against the CAZy database (release 08062022) and HMMER3 against the run_dbcan v3.0.7 library [28]. BlastP alignments were filtered with an e-value of 1e-50 and 95% coverage of both the query and subject sequence. CAZymes were further scanned with cupp v.4.0.0 [29] for EC or subfamily annotations. Proteins that were not classified as either extracellular or membrane-bound with DeepLoc2 [30] and lacked transmembrane helices after a scan with DeepTMHMM [31] were considered intracellular and were excluded from downstream analysis.

### Proteomic data analysis

Identification and relative quantification were performed using Proteome Discoverer version 2.4 (Thermo Fisher Scientific, Waltham, MA, USA). Data were matched against the proteome of *T. terrestris* LPH172 [13]. Database matching was performed using Mascot (v. 2.5.1, Matrix Science), with a precursor tolerance of 5 ppm and a fragment ion tolerance of 0.6 Da. Tryptic peptides were accepted if they contained no missed cleavages; methionine oxidation was set as a variable modification; whereas cysteine methylthiolation, TMTpro on lysine, and peptide N-termini were set as fixed modifications. Percolator was used for peptide spectrum match validation with a strict false discovery rate (FDR) threshold of 1%. For quantification, TMT reporter ions were identified in MS3 HCD spectra with 3 mmu mass tolerance. The SPS Mass Match threshold was set to 45% and only unique peptides were used for relative quantification.

Proteins with an FDR threshold of 1% and quantified in at least two biological replicates using two substrates were kept for quantitative analysis. The DEP R package [32] was used to explore the overlap between detected proteins in each substrate and check the link between missing values and protein abundance. Protein abundances were subjected to median normalization and missing values were imputed with the MinProb function of the imputeLCMD package. Differential protein abundance between substrates was assessed using the LIMMA moderated test statistic [33] with a Benjamini–Hochberg FDR of 5% and a minimum log2 fold change greater than 1.

Intracellular proteins were excluded from network analysis. Z-scores of imputed protein abundances were calculated for each protein by subtracting the mean protein abundance across all samples and then dividing by the standard deviation. They were then used for downstream analyses. Network analysis was performed using the weighted correlation network analysis (WGCNA) R package v1.72–1 [34] to group proteins with similar expression patterns. Correlation of protein abundances was calculated using the biweight midcorrelation, a robust alternative to Pearson correlation [35], and was raised to a power of 18 to create a weighted signed network. The topological overlap matrix of the network was used to group the proteins in modules, with a minimum of 10 proteins per module. One-sided Fisher's exact test was used to assess the enrichment of particular enzyme families within a module when compared to the entire network population, applying a p-value threshold of 5%. Module expression profiles were visualized using the R package PloGO2 [36].

### Enzymatic hydrolysis of steam-pretreated spruce

To evaluate the hydrolytic efficiency of enzymes secreted by *T. terrestris*, the fungus was first grown on STEX_180ºC/auto_ for 4 days. The choice of material and growth period was based on proteomic analysis and overtime activity measurements. Five different flasks were prepared, of which one was not inoculated and kept as control. Four days after inoculation, the supernatant containing the STEX_180ºC/auto_ secretome was collected, centrifuged to separate solid and liquid fractions, and concentrated (~ 20 times) using the Vivaspin 10 kDa insert (Pall). At the end of the concentration step, enzymatic activity was checked (qualitative evaluation, data not shown) using the endo-xylanase assay. The protein concentration was determined to be 0.03 g L^−1^ with the Bradford assay. Enzymatic hydrolysis was assessed in triplicates, in 2-mL screw-cap tubes with a total reaction weight of 1.8 g. The substrate chosen for enzymatic hydrolysis was STEX_210ºC/HAc_ (2% w/w DM) suspended in 0.15 M acetate buffer (pH 5). The reaction mixture was autoclaved at 121 °C for 20 min, after which sterile-filtered enzyme cocktails were added aseptically: 5 FPU g^−1^ DM for Celluclast® (54 FPU mL^−1^; 43.1 g L^−1^) and 5 U g^−1^ DM for Novozym188 (28 U mL^−1^; 34.0 g L^−1^). The STEX_180ºC/auto_ secretome was supplemented to the enzymatic cocktails at 1.01 mg protein g^−1^ DM, which corresponded to 10% (w/w) of the protein content loaded with Celluclast® and Novozym188. Enzymatic hydrolysis was performed using different combinations of Celluclast®, Novozym188, and the STEX_180ºC/auto_ secretome: 10% (w/w) STEX_180ºC/auto_ secretome; 5 FPU g^−1^ DM of Celluclast®; 5 FPU g^−1^ DM of Celluclast® + 10% (w/w) STEX_180ºC/auto_ secretome; 5 FPU g^−1^ DM of Celluclast® + 5 FPU g^−1^ DM of Novozym188; 5 FPU g^−1^ DM of Celluclast® + 5 FPU g^−1^ DM of Novozym188 + 10% (w/w) STEX_180ºC/auto_ secretome. Enzymatic hydrolysis was carried out at 40 °C for 48 h in an oven with end-over-end rotation (Big S.H.O.T III™; Boekel Scientific, Feasterville, PA, USA) and constant rotation at 25 rpm. To terminate the reaction, the mixture was boiled for 10 min. The samples were then either stored at − 20 °C or processed directly for sugars release analysis. The release of glucose, mannose, and xylose was measured by isocratic high-performance anion-exchange chromatography with pulsed amperometric detection (ICS-5000; Dionex, Sunnyvale, CA, USA) using a Carbopac PA1 column as described elsewhere [37]. The samples were centrifuged for 5 min at 13,000 rpm, and the supernatants were filtered through 0.22-µm syringe-driven filters before storage at 4 °C until analysis.

## Results and discussion

In the present study, the filamentous fungus *T. terrestris* was grown on differently steam-pretreated spruce biomass. First, the effect of different sugars as CAZyme inducers was evaluated to determine whether it was possible to speed up fungal growth by inducing lignocellulolytic enzymes in the preculture stage. Once optimal preculture conditions were identified, the impact of varying composition and structure of spruce materials on the fungal secretome was assessed. Finally, the most heterogeneous secretome, inducing the highest enzymatic activities, was identified and used as a tailor-made supplement to enhance a commercially available enzyme cocktail for the saccharification of a steam-pretreated spruce material.

### Time-course of enzyme induction by *T. terrestris* grown on spruce

Inducers such as hexoses, pentoses, sorbose, and sophorose have an impact on the enzyme secretion pattern [38]. In the present study, glucose, xylose, arabinose, cellobiose, and maltose were tested as enzyme inducers in *T. terrestris* precultures. Samples were taken at 3, 4, and 6 days to measure endo-cellulase, endo-xylanase and endo-mannanase activities. Arabinose was selected as carbon source for the precultures due to the high endo-xylanase activity detected after 3 days (Figure S1). Endo-mannanase and endo-cellulase activities were below the detection limit (data not shown).

**Fig. 1:**
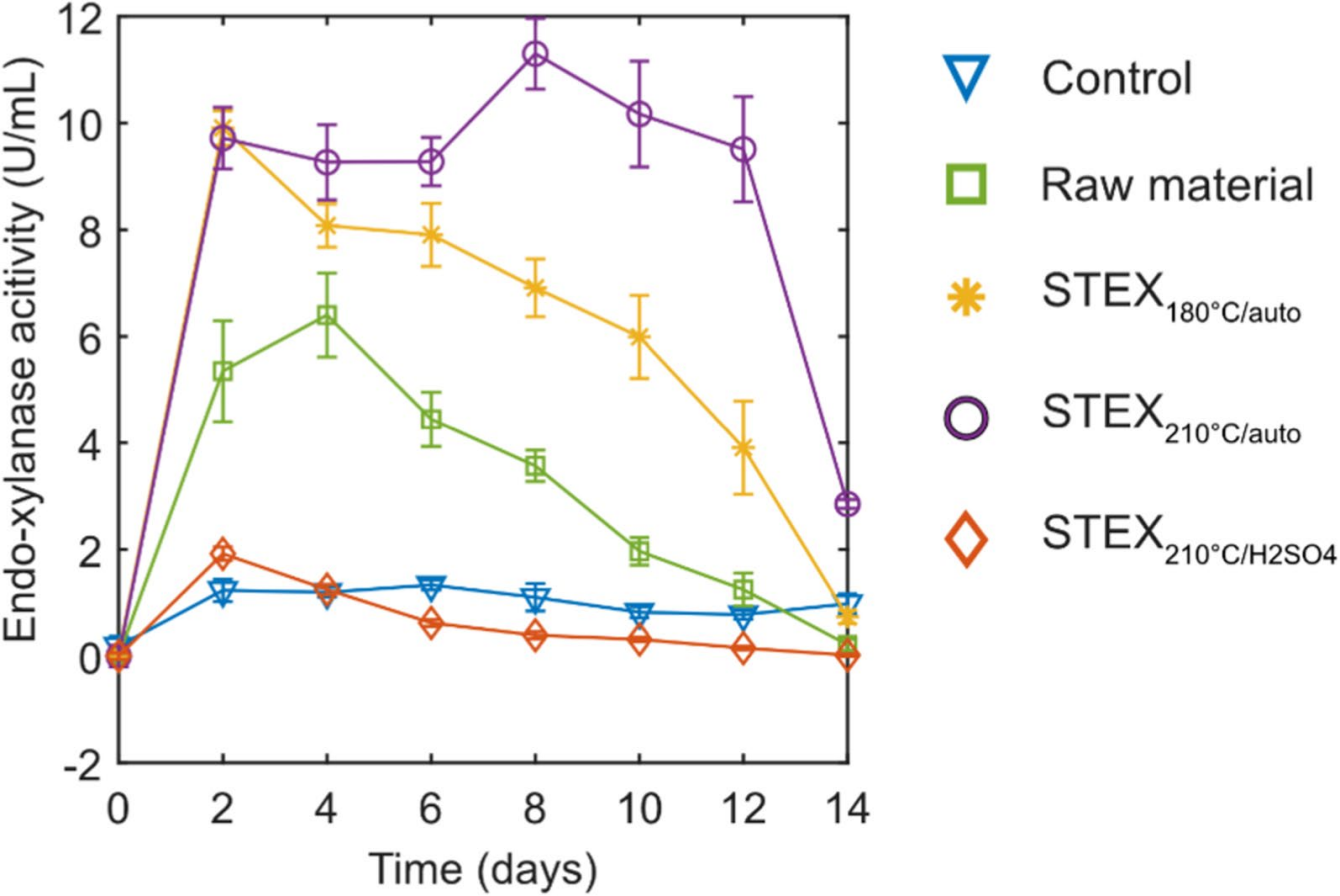
Endo-xylanase activity of *T. terrestris* during growth on steam-pretreated spruce biomass. Milled raw material, STEX_180°C/auto_, STEX_210°C/auto_, and STEX_210°C/H2SO4_ were used to grow *T. terrestris* for 14 days. Endo-xylanase activity was measured every other day. Control cultures contained no lignocellulosic material. Data represent the mean ± standard deviation of triplicate measurements

Time-course measurements revealed how enzymatic activity changed during cultivation of *T. terrestris* on spruce. The fungus was grown for 14 days on untreated and steam-pretreated spruce. Endo-mannanase, laccase, and peroxidase activities were not detected throughout cultivation in any of the samples. Endo-cellulase was detected at low levels (< 0.03 U mL^−1^) when the fungus was grown on STEX_180°C/auto_ and STEX_210°C/auto_ (data not shown). Endo-xylanase activity was substrate-dependent and significant differences were found in the different cultivations (Fig. 1). After 2 days of growth on STEX_180°C/auto_, STEX_210°C/auto_, untreated material, and STEX_210°C/H2SO4_, endo-xylanase activity reached 9.9 U mL^−1^, 9.7 U mL^−1^, 5.4 U mL^−1^, and 1.9 U mL^−1^, respectively. Between 4 and 12 days, activity was almost constant during growth on STEX_210°C/auto,_ but decreased steadily when the fungus was cultivated on STEX_180°C/auto_. On the untreated material, the endo-xylanase activity reached the highest value of 6.4 U mL^−1^ at 4 days and then constantly decreased till 14 days. On STEX_210°C/H2SO4_, endo-xylanase activity remained equal or lower than in the control (absence of lignocellulosic material). The higher endo-xylanase activity measured when *T. terrestris* was grown on STEX_180°C/auto_ and STEX_210°C/auto_ can be explained by different chemical composition (Table S1) and structural features resulting from pretreatment. Steam pretreatment causes hemicellulose degradation, while lignin is depolymerized and recondensed on the surface [15, 39]. As a result, pretreated materials are easier to degrade compared to untreated biomass. In the case of *T. terrestris* grown on STEX_210°C/H2SO4_, endo-xylanase activity likely dropped due to low amounts of xylan, which are probably hard to degrade [20].

### Secretome analysis and comparison to the *T. terrestris* genome

The secretomes of *T. terrestris* grown in the aforementioned materials for 4 days were isolated and analyzed. The choice of the 4-day time point was based on the endo-xylanase activity (Fig. 1), which represented a good compromise in activity values among the different materials tested. Fungal secretomics is a powerful tool to gain a deeper view of enzyme activities found beyond the detection limits of applied enzymatic assays. Of the 509 proteins identified using Proteome Discoverer with a false discovery rate (FDR) of 1%, 312 were reliably quantified and 36.7% of these were predicted to be intracellular. A total of 111 non-intracellular CAZymes were quantified, corresponding to 35% of the total non-intracellular CAZyme content of the *T. terrestris* genome. Notably, for this study, the *T. terrestris* proteome has been functionally re-annotated with a more recent version of the CAZy database. This led to the annotation of 16 glycoside hydrolases (GHs), 11 auxiliary activities (AAs) and 1 carbohydrate-binding module (CBM) more compared to the original report [13].

**Fig. 2:**
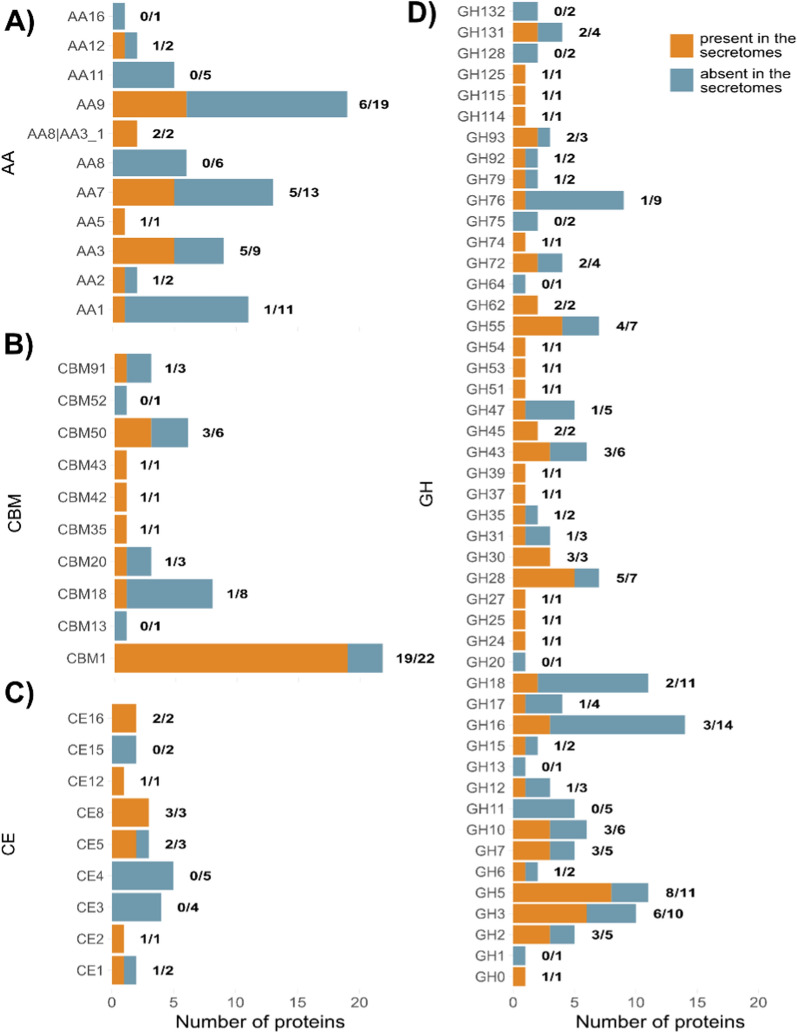
CAZyme families present in the genome (blue bars) and in the secretome (orange bars) of *T. terrestris*. **A** Auxiliary activities, **B** carbohydrate-binding modules, **C** carbohydrate esterases, and **D** glycosyl hydrolases

AA family members were the least represented in the secretomes (Fig. 2A). None of the six AA8 iron reductases were detected, and only one laccase (AA1) was expressed. However, both AA8|AA3_1 cellobiose dehydrogenases (CDHs), approximately half of the AA3 flavoproteins, and AA7 glucooligosaccharide oxidases were quantified. Among lytic polysaccharide monooxygenases (LPMOs), six members of the AA9 family were quantified, three of which also contained a CBM1. Conversely, none of the five AA11 chitin-active LPMOs were detected, which could be explained by the absence of chitin in spruce.

Of the 44 CBM-harboring proteins encoded in the genome of *T. terrestris*, 27 were quantified in this study (Fig. 2B). The CBM1 family was the most abundant, with nearly all members present (19/22), along with three chitinases harboring the CBM50 domain. In contrast, the CBM18 chitin-binding family, the second most abundant family in the genome, was almost completely absent, as expected due to the absence of chitin in spruce.

Ten of the 23 carbohydrate esterase (CE) genes (Fig. 2C) that primarily target acetyl groups on poly- or oligosaccharides were detected. Both acetylesterases of the CE16 family were present, along with all pectin esterases of the CE8 family, despite the absence of pectin in the tested materials. In contrast, the two most abundant CE families in the genome, CE3 and CE4, were absent from all secretomes. These two CE families potentially target xylan, chitin, and peptidoglycan.

GHs account for 51% of CAZymes in the genome of *T. terrestris*. Under the tested conditions, 47% of GH CAZymes were detected in the secretomes (Fig. 2D). Notably, GH5 family was the most abundant with five endo-beta-1,4-glucanases, two endo-beta-1,4-mannanases, and an endo-beta-1,6-galactanase. The GH3 family, which contains beta-glucosidases and 1,4-beta-xylosidases, ranked second. The GH16 family encoding chitin, xyloglucan, and carrageenan glycosylases/transferases is the most abundant in the genome of *T. terrestris*, but only 3 of 14 enzymes were expressed on the various spruce materials. Conversely, the GH76 and GH18 families were notably absent, as only a few members were present. The GH76 family consists of enzymes acting on alpha-mannan and alpha-glucan; whereas the GH18 family includes enzymes responsible for peptidoglycan and chitin degradation.

Arntzen et al. [40] used label-free proteomics to study the secretome of *Myceliophthora thermophila* DSM 1799 grown on spruce for 3 days. *M. thermophila* is a thermophilic ascomycete of the Sordariales order, the same as *T. terrestris*, and its annotated genome is available in MycoCosm [41]. Almost all members of the CBM1 family were present in both fungal secretomes. For *M. thermophila*, these proteins were present when the fungus was grown in bagasse and birch, but were absent from cultivation in glucose, indicating that they were constitutively involved in lignocellulose degradation and were not spruce-specific. Conversely, lignin-active auxiliary enzymes, such as laccases and peroxidases, were conspicuously absent or underrepresented in the secretomes of both fungi when grown on spruce. While 11 of the 15 AA enzymes detected in the secretome of *M. thermophila* cultivated on spruce were LPMOs belonging to the AA9 family; *T. terrestris* exhibited a distinct profile, with LPMOs constituting only a minor fraction of AA enzymes. Additionally, *M. thermophila* lacked two specific AA8|AA3_1 CDHs with a cytochrome domain that were present in the *T. terrestris* secretome and did not secrete any oxidase of the AA7 family. *T. terrestris* secreted a total of 82 different GHs, which is almost double the amount secreted by *M. thermophila* when growing on spruce (42 GHs). Particularly, *M. thermophila* secreted fewer members of the GH2, GH3, GH16, and GH30 families compared to *T. terrestris*; whereas GH5, GH7, and GH10 representatives were present in both fungi to a similar extent. *T. terrestris* secreted a wider range of CEs than *M. thermophila* when grown on spruce but, interestingly, *M. thermophila* secreted CE1, CE3, and CE10 when growing on bagasse and birchwood, indicating substrate-specific regulation of enzyme expression.

### The *T. terrestris* secretome is affected by the composition and structure of spruce

Among the 198 quantified non-intracellular proteins, 188 were detected in the secretomes of *T. terrestris* grown on all three pretreated spruce materials; whereas 21 proteins were absent from the secretome of fungi cultivated on untreated biomass. The Pearson correlation coefficient among three biological replicates exceeded 92% under all conditions, indicating high similarity between biological replicates. Principal component analysis (PCA) showed that the biological replicates formed distinct clusters and the primary factor driving the variation in protein expression was the severity of spruce pretreatment (Figure S1). Interestingly, PCA revealed that the secretome of *T. terrestris* grown on STEX_210°C/auto_ had a closer resemblance to that of fungi cultivated on STEX_210°C/H2SO4_ than STEX_180°C/auto_. STEX_210°C/auto_ and STEX_210°C/H2SO4_ secretomes aligned more closely along the x-axis of the PCA plot, which is the axis that captures most of the observed variance. Such pattern was surprising, given that the composition (Table S1) and cellulose ultrastructure (crystallite size) of STEX_210°C/auto_ was more similar to STEX_180°C/auto_ than to STEX_210°C/H2SO4_ [20].

**Fig. 3:**
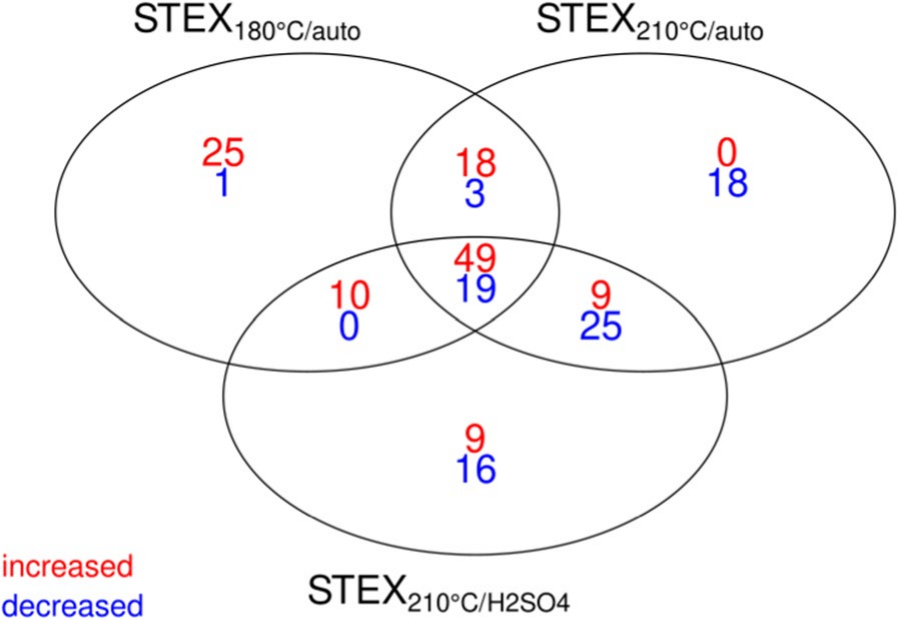
Venn diagram of non-intracellular proteins with differentially increased (red) or decreased (blue) abundance in pretreated spruce compared to untreated biomass

Differential protein abundance across substrates revealed that 120 out of 198 non-intracellular proteins were more abundant in the secretome of *T. terrestris* cultivated on one or more of the pretreated materials than raw biomass (Fig. 3). In contrast, only 82 proteins displayed increased abundance in the secretome of *T. terrestris* cultivated on raw biomass compared to the secretome of at least one of the pretreated materials. Most proteins were abundant when the fungus was grown on STEX_180°C/auto_ and, as anticipated by PCA, they followed a similar expression pattern in STEX_210°C/auto_ and STEX_210°C/H2SO4_ samples.

### Clustering of co-regulated proteins with the WGCNA package

**Fig. 4:**
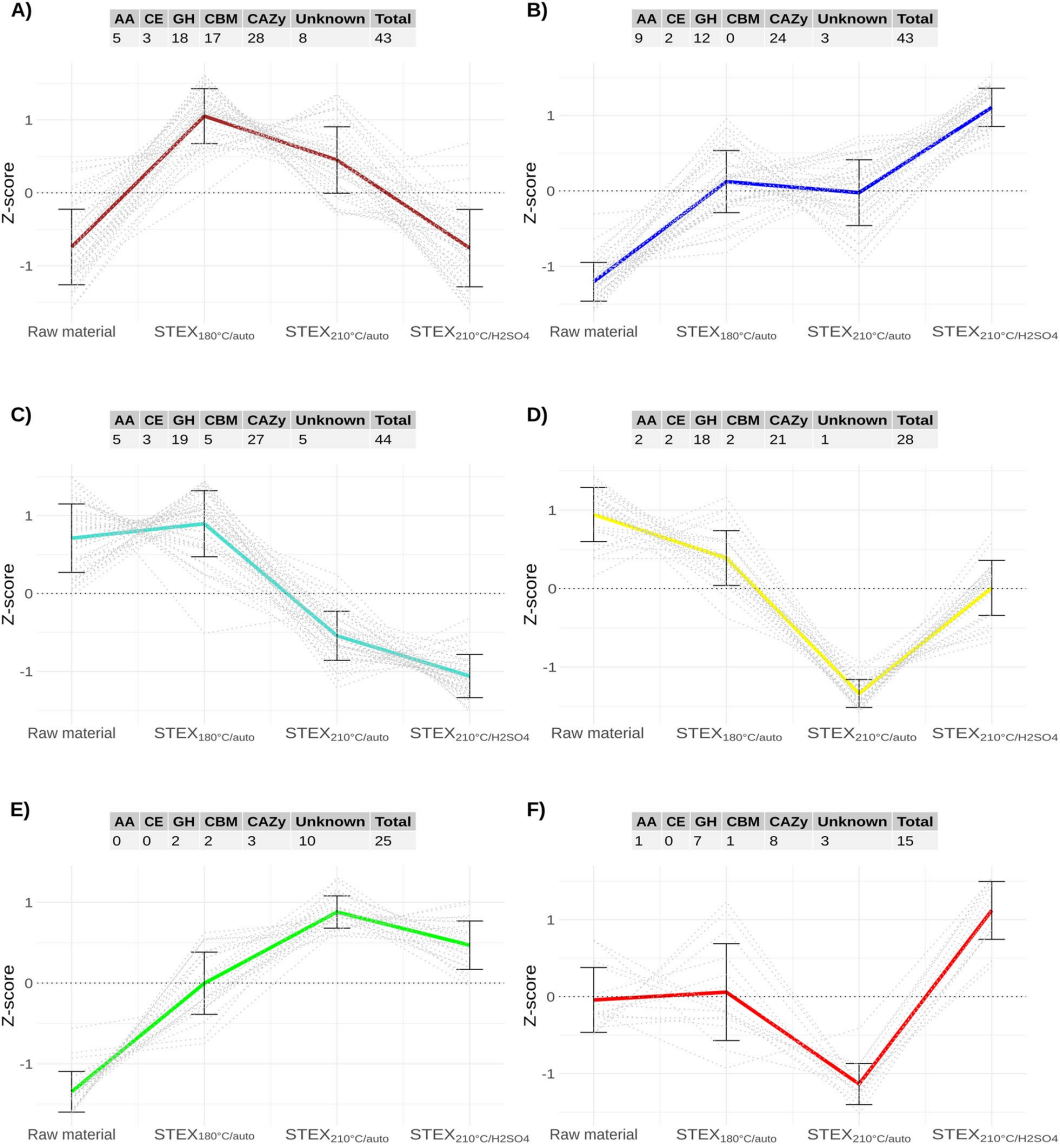
Average z-scores of protein abundance and number of proteins from the different classes of enzymes for each module (clusters of proteins showing similar abundance across the samples). **A** Brown, **B** blue, **C** turquoise, **D** yellow, **E** green, and **F** red. Z-scores for each individual protein module member are plotted as dashed lines

To gain deeper insights into the regulation of the secretome and its response to spruce pretreatment, network analysis using the WGCNA R package was performed. The analysis focused on non-intracellular proteins, allowing the identification of clusters of co-regulated proteins and revealed their response to the different pretreatments. WGCNA calculates pairwise correlations between proteins using protein abundances across all samples and then performs clustering based on topology overlap similarity (TOM) as the measured distance. The 198 non-intracellular proteins subjected to WGCNA were clustered into six distinct groups, referred to as modules. Each module contained proteins with a similar secretion pattern on the different materials used and displayed a distinct expression profile (Fig. 4). Each module was assigned a different color for visualization and reference purposes (brown, blue, turquoise, yellow, green, and red). The number of proteins included in the modules ranged from 15 (red) to 44 (turquoise) (Table S2).

The brown module encompassed enzymes that were secreted mainly in the presence of the two autocatalyzed materials, and especially STEX_180°C/auto_. Of the 28 CAZymes identified, 17 were enriched in CBMs-containing enzymes, as confirmed by the statistical Fisher’s exact test. Specifically, 13 enzymes harbored a CBM1, hinting at their potential role in cellulose degradation [42]. The brown module comprised also 3 AA9 LPMOs, 2 of which possessed a CBM1 and were particularly abundant in the secretome of the fungus grown on STEX_180°C/auto_ compared to all the other substrates.

The blue module contained most of the AA enzymes, but none of the AA9 LPMOs and the level of secretion increased with increasing pretreatment severity, peaking in STEX_210°C/H2SO4_ (Fig. 4). Interestingly, this module featured both predicted extracellular lactonases, along with a CDH and four AA7 glucooligosaccharide oxidases. Lactonases may be acting synergistically with CDHs or glucose oxidases as they catalyze the hydrolysis of sugar lactones generated by CDH or LPMOs to aldonic acids [43]. Sugar lactones could also inhibit β-glucosidases and, therefore, the presence of lactonases might boost biomass hydrolysis [44]. This module encompassed four oxidases belonging to the AA3 family and four oxidases from the AA7 family. It also included a CE16 acetylesterase, three GH3 beta-glucosidases, a GH30_7 glucuronoxylanase, a xylan alpha-1,2-glucuronidase, a GH10 beta-xylanase, an alpha-L-arabinofuranosidase, and a GH79 glucuronidase.

The proteins in the turquoise module became less abundant with increasing severity of pretreatment, as indicated by the highest amounts in the STEX_180°C/auto_ sample. Among CAZymes, this module displayed the highest number of GHs compared to the other modules.

The yellow module clustered proteins that were abundant in the secretome of the fungus grown on untreated biomass and with the lowest levels in STEX_210°C/auto_. This module contained 28 proteins, including 18 GHs, and the sole detected AA1 laccase. GHs included 2 GH72 beta-glucanases, a CBM42|GH54 alpha-L-arabinofuranosidase, and 2 exo-alpha-L-1,5-arabinanases.

The green module comprised 25 proteins, whose secretion levels were significantly low when the fungus was cultivated on raw material but increased with the severity of pretreatment, peaking in STEX_210°C/auto_. This module contained only 3 CAZymes and it was significantly enriched in proteins of unknown function (9 out of 27; as confirmed by Fisher's exact test).

The red module included only 15 proteins, of which 8 were CAZymes. Proteins belonging to this module exhibited an intriguing secretion pattern in response to the different substrates. Protein abundance remained relatively stable in the raw material and STEX_180°C/auto_; whereas a significant drop was seen in STEX_210°C/auto_ and rise in STEX_210°C/H2SO4_.

Overall, network analysis revealed that there are classes of enzymes with similar expression pattern across secretomes, suggesting a potentially synergistic activity (i.e., blue module). The abundance of the different classes within one module changed across materials even though they were all differently steam-treated spruce. This could probably mean that the secretomes composition is affected not only from the composition, but also from the structure of the biomass, indicating that the different materials required different enzyme ratios to be degraded. Indeed, the most heterogenous secretome resulted from growth on STEX_180°C/auto_. This material presents a more complex structure due to the highest percentage of hemicellulose compared to the other steam-treated spruce biomass [20] with a better accessibility than untreated biomass due to the pretreatment.

### Supplementation of an industrial cellulolytic cocktail with *T. terrestris* secretomes for the hydrolysis of STEX_210°C/HAc_

The hydrolytic efficiency of enzymes secreted by *T. terrestris* grown on steam-pretreated spruce was tested on STEX_210°C/HAc_. The *T. terrestris* secretomes were hypothesized to represent an adaptation of the fungus that facilitated the release of sugars from spruce. Therefore, the fungus was grown for 4 days on STEX_180°C/auto_ and the secreted enzymes were evaluated as a supplement for the commercial mixtures Celluclast® and Novozym188 in different combinations. STEX_180°C/auto_ was selected as the substrate for enzyme production because the resulting secretome had the largest number of CAZymes compared to the other secretomes. As confirmed by WGCNA, the vast majority of LPMOs, CEs, and CBMs could be detected in the secretome of the fungus cultivated on STEX_180°C/auto_.

**Fig. 5:**
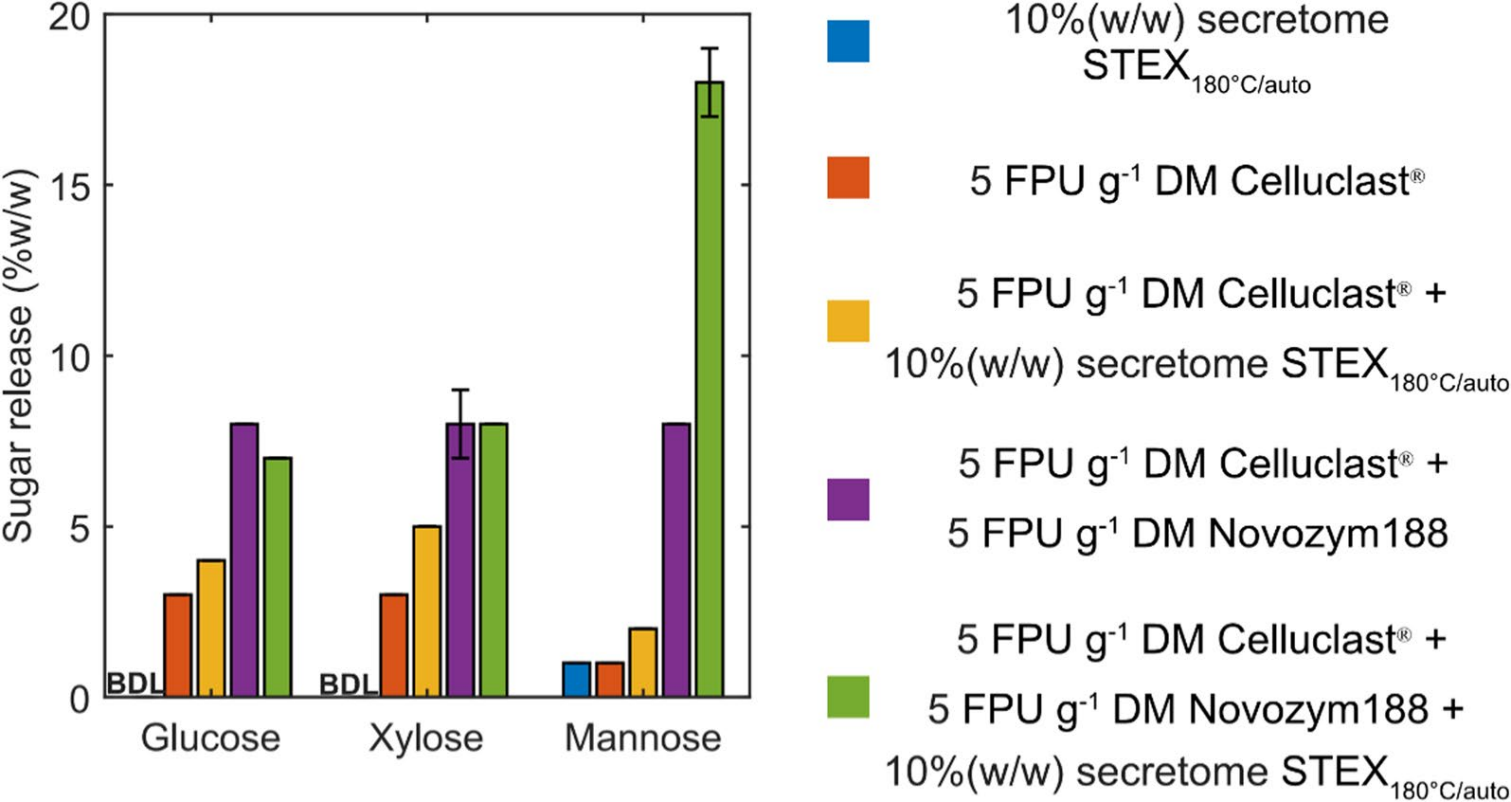
Enzymatic hydrolysis of STEX_210°C/HAc_ using different combinations of commercial enzymes and the *T. terrestris* secretome. Release of glucose, xylose, and mannose was measured after 48 h of hydrolysis using: only the enzymes secreted by *T. terrestris* grown on STEX_180°C/auto_ (blue bars), Celluclast (orange bars), Celluclast supplemented with the secreted enzymes from *T. terrestris* (yellow bars), Celluclast + Novozym188 (purple bars), and Celluclast + Nozym188 supplemented with the secreted enzymes from *T. terrestris* (green bars)*.* Data represent the mean ± standard deviation of triplicate measurements. Where error bars are not represented, they are below 0.5%

The hydrolytic efficiency of the secreted enzymes alone (blue bar, Fig. 5), which resulted in 1% mannose being released, provided the reference point for subsequent evaluations. The sugars released with and without supplementation of the commercial mixtures with the secreted enzymes were then compared (Fig. 5). Celluclast® supplemented with the secretome achieved a 1.3-, 1.7-, and twofold increase in glucose, xylose, and mannose release, respectively, compared to Celluclast® alone. When supplementing the secreted enzymes to Celluclast® + Novozym188, a 2.3-fold increase in mannose release was observed; whereas glucose and xylose release did not improve further. The observed increase in mannose release can be attributed to the high levels of GH5_7 in the secretome. Indeed, the secretome obtained from STEX_180°C/auto_ showed the highest level of mannosidases compared to the other secretomes.

## Conclusions

In the present study, substrate-induced changes in the secretome of *T. terrestris* grown on differently steam-pretreated spruce biomass were investigated. As a result of different pretreatment conditions, spruce biomass presented diverse hemicellulose content and structural characteristics. Overall, 120 out of the total 198 non-intracellular proteins exhibited increased abundance in at least one of the pretreated materials when compared with untreated biomass. To better understand the regulation of the secretome and its response to spruce pretreatment, the 198 non-intracellular proteins were subjected to WGCNA and clustered into six distinct modules. These modules clustered enzymes classes with similar secretion patterns as response to the different pretreated materials used. Most enzymes overlapped across the different secretomes, although some classes were more abundant upon growth on certain substrates and probably contributed the most to saccharification of spruce biomass. The secretome of *T. terrestris* was most heterogeneous when the fungus was grown on STEX_180°C/auto_. Therefore, the hydrolytic efficiency of enzymes secreted by *T. terrestris* cultivated on STEX_180°C/auto_ was tested on a different steam-pretreated material (STEX_210°C/HAc_). Supplementing the secreted enzymes to Celluclast® + Novozym188 increased by 2.3-fold the release of mannose, which could be explained by the high levels of GH5_7 in the fungal secretome. As shown here, these tailor-made secretomes can enhance spruce saccharification when combined with commercial enzyme cocktails, thereby opening new possibilities for improved enzymatic hydrolysis via targeted enzyme supplementation.

## Supplementary Information

Additional file 1.

## Data Availability

Mass spectrometry proteomics data along with an extended description of the experimental procedure have been deposited to the ProteomeXchange Consortium via the PRIDE partner repository, with dataset identifiers PXD047620 and 10.6019/PXD047620. All the other data generated or analyzed during this study are included in this published article and its additional files.
