# Supplementary material for: Elucidating Thermothielavioides terrestris secretome changes for improved saccharification of mild steam-pretreated spruce
Source: Biotechnol Biofuels Bioprod. 2024 Oct 5;17:127. doi: 10.1186/s13068-024-02569-3 (PMC11456254; doi:10.1186/s13068-024-02569-3)
Supplement: Supplementary file 1 — Additional file 1. [file 13068_2024_2569_MOESM1_ESM.docx]

**Supplementary material**

Table S1: Compositional analysis of lignocellulosic compounds in spruce before and after pretreatment. Data represent mean values ± standard deviation. Analyses were performed in triplicate. The glucan amount corresponds to the amount of glucose from cellulose and from galactoglucomannan.

|  | **Raw material**  **(%w/w DM)** | **STEX_180°C/auto_  (%w/w DM)** | **STEX_210°C/auto_**  **(%w/w DM)** | | **STEX_210°C/HAc_ (%w/w DM)** | **STEX_210°C/H2SO4_**  **(%w/w DM)** |
| --- | --- | --- | --- | --- | --- | --- |
| Glucan | 45.6 ± 0.7 | 49 ± 1 | 52 ± 2 | 60.5 ± 0.3 | | 61.0 ± 0.1 |
| Xylan | 5.0 ± 0.1 | 4.9 ± 0.6 | 2.4 ± 0.1 | 1.7 ± 0.0 | | 0.7 ± 0.0 |
| Arabinan | 0.5 ± 0.0 | 0.0 ± 0.0 | 0.0 ± 0.0 | 0.0 ± 0.0 | | 0.0 ± 0.0 |
| Galactan | 1.7 ± 0.1 | 1.5 ± 0.1 | 0.2 ± 0.0 | 0.1 ± 0.0 | | 0.1 ± 0.0 |
| Mannan | 12.1 ± 0.1 | 10.7 ± 0.6 | 4.1 ± 0.2 | 1.2 ± 0.0 | | 0.6 ± 0.0 |
| ASL | 2.6 ± 0.1 | 2.3 ± 0.1 | 1.4 ± 0.0 | 2.9 ± 0.1 | | 2.7 ± 0.1 |
| AIL | 32.4 ± 0.1 | 34.4 ± 0.6 | 41.4 ± 0.4 | 36.0 ± 0.1 | | 38.2 ± 0.4 |
| Ash | BDL | BDL | BDL | BDL | | BDL |
| Recovery | 100 ± 1 | 103 ± 2 | 101 ± 1 | 103 ± 1 | | 103 ± 1 |

AIL, acid-insoluble lignin; ASL, acid-soluble lignin; BDL, below detection limit.

Table S2 Number of proteins per cazy family in each module.

|  | blue | brown | green | red | turquoise | yellow | quant | detected | genome |
| --- | --- | --- | --- | --- | --- | --- | --- | --- | --- |
| AA | 9 | 5 |  | 1 | 5 | 2 | 22 | 26 (36,6%  ) | 71 |
| AA9 |  | 3 |  |  | 2 | 1 | 6 | 7 (36,8%  ) | 19 |
| CE | 2 | 3 |  |  | 3 | 2 | 10 | 11 (47,8%  ) | 23 |
| GH | 12 | 18 | 2 | 7 | 19 | 18 | 76 | 83 (51,2%  ) | 162 |
| CBM |  | 17 | 2 | 1 | 5 | 2 | 27 | 29 (65,9%  ) | 44 |
| CBM1 |  | 13 | 2 | 1 | 3 |  | 19 | 20 (90,9%  ) | 22 |
| all_cazymes | 24 | 28 | 3 | 8 | 27 | 21 | 111 | 126 (39,4%  ) | 320 |
| uncharacterized | 2 | 7 | 9 | 3 | 5 | 1 | 27 | 46 | nan |
| proteases | 3 | 1 |  |  | 2 | 2 | 8 | nan | nan |
| **all_proteins** | **43** | **43** | **25** | **15** | **44** | **28** | **198** | **255** | **nan** |

**S1 Total fungal biomass**

To measure the fungal biomass, the 50 mL falcon tubes used for the growth were centrifuged (5000 rpm for 5 min) to divide the biomass from the liquid fraction. After centrifugation, the supernatant was discarded, the biomass was washed twice by resuspending it in milliQ water and centrifuged again. Then the tubes were put in the oven (50ºC) until the weight of the tube was constant (usually after 18 h).


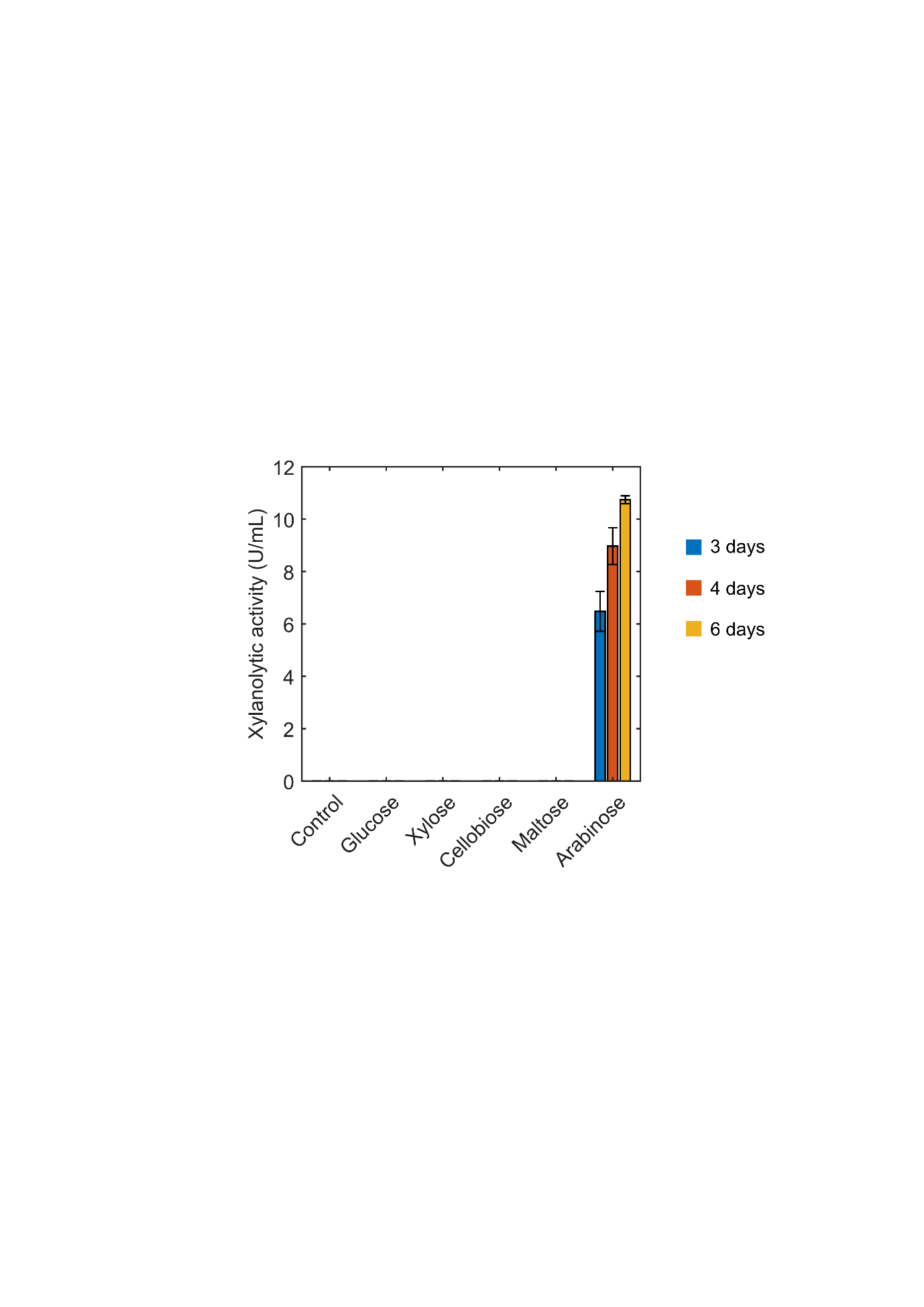


**Figure S1. Xylanolytic activity of *T. terrestris* after 3, 4 and 6 days on different carbon sources.** Data represent the mean ± standard deviation of triplicate measurements.
